# Growth Rate Analysis of an Untreated Glomus Vagale on MRI

**DOI:** 10.1155/2016/8756940

**Published:** 2016-03-17

**Authors:** Jeffrey Tzu-Yu Wang, Allen Yu-Yu Wang, Sheila Cheng, Lavier Gomes, Melville Da Cruz

**Affiliations:** ^1^Department of Radiology, Westmead Hospital, University of Sydney, Sydney, NSW 2145, Australia; ^2^Department of Otolaryngology, Westmead Hospital, University of Sydney, Sydney, NSW 2145, Australia

## Abstract

Paragangliomas are slow growing, hypervascular neuroendocrine tumors that develop in the extra-adrenal paraganglion tissues. Paraganglioma involving the vagus nerve ganglia is termed glomus vagale. The slow growth of head and neck paragangliomas especially in the absence of symptom may obviate the necessity for any active intervention, in which case, a “wait and scan” policy is implemented involving long-term clinical and radiologic follow-ups. We present a case of a 71-year-old female with an untreated left glomus vagale who underwent a conservative “wait and rescan” plan of management and the tumor was observed with 8 serial MRI scans over a period of 7.4 years. A growth rate analysis was conducted which demonstrated a slow growth. A literature review of radiologic studies examining the natural history of head and neck paragangliomas was also performed.

## 1. Introduction

Paragangliomas are slow growing, hypervascular neuroendocrine tumors that develop in the extra-adrenal paraganglion tissues. In the head and neck region they account for 0.6% of all tumors and are labeled by their anatomic site of origin: vagus nerve ganglia (glomus vagale), carotid body at the bifurcation of common carotid artery (carotid body tumor), jugular bulb (glomus jugulare), and tympanic plexus (glomus tympanicum). Approximately 80% of head and neck paragangliomas are either carotid body tumors or glomus jugulare [1].

The management options include surgical resection, conventional radiation therapy, stereotactic radiosurgery, permanent embolization, a combination of these modalities, and observation. Surgery is the only curative treatment but it may be complicated by significant postoperative morbidity particularly in larger tumors [1]. There has been a shift in the treatment paradigm to a more conservative approach. Improved knowledge, experience, and recognition of a low incidence of malignancy have allowed individualization of management [2]. The slow growth of these tumors, especially in the absence of symptom, may obviate the necessity for any active intervention, in which case, a “wait and scan” policy is implemented involving long-term clinical and radiologic follow-ups. The imaging modalities used in the radiologic follow-up are computed tomography (CT) or magnetic resonance imaging (MRI) [3].

In the existing literature, there have been four radiologic studies which have examined the natural history of head and neck paragangliomas [2, 4–6]. Further robust data concerning the growth of these tumors is required which will assist clinicians in treatment planning. In head and neck paragangliomas, tumor growth and surgical resection can lead to disabling loss of cranial nerve (CN) functions; therefore having an insight into the natural history is essential for the development of treatment strategies. In this paper we present a case of an untreated left glomus vagale who underwent 8 serial MRI scans over a period of 7.4 years, and the growth rate analysis of the tumor.

## 2. Case Presentation

A 71-year-old female presented with mild pharyngeal discomfort. Clinical examination revealed medial displacement of the left tonsil associated with transmitted pulsation of the surrounding pharyngeal wall. No cranial nerve palsy was evident. MRI showed a well-defined mass in the left parapharyngeal space. *T*_1_ weighted sequences demonstrated low signal intensity with multiple voids. On *T*_2_ weighted sequences the mass was of high signal intensity and revealed extensive enhancement following administration of gadolinium. Radiologic features were consistent with glomus vagale.

In light of her minimally disabling symptoms and the tumor being located on the side of her only seeing eye, the patient underwent a conservative “wait and rescan” plan of management. The tumor was observed with 8 serial MRI scans over a period of 7.4 years since 2006. This allowed a linear regression model to estimate the growth rates of its maximum axial dimension, maximum axial area, and volume.

**Figure 1:**
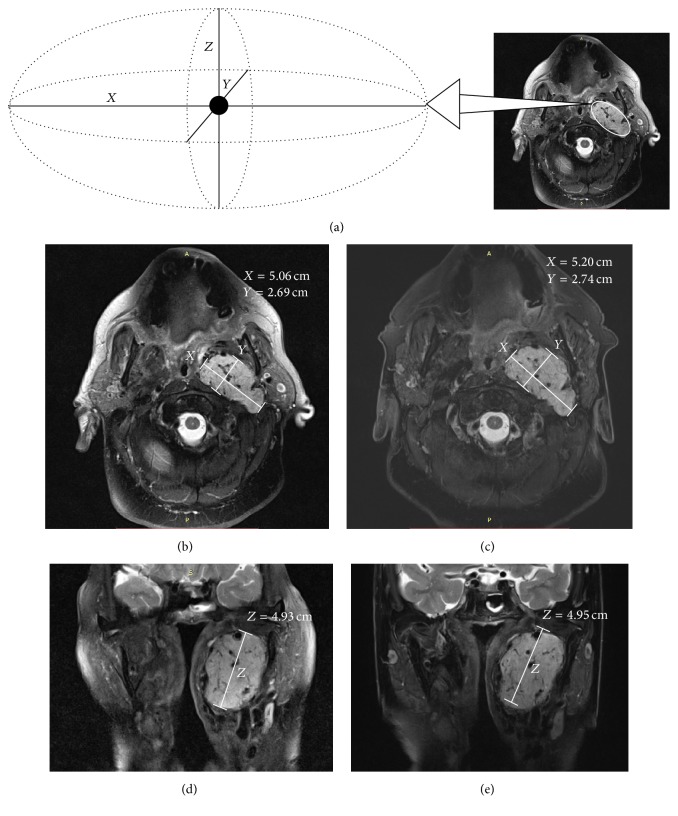
(a) The untreated left glomus vagale was considered to have an ellipsoid shape. The 3 perpendicular dimensions in the anteroposterior (*X*), mediolateral (*Y*), and craniocaudal (*Z*) directions were used to calculate the maximum axial area and volume. ((b)–(e)) Serial MRI scans (*T*_2_ fat saturation suppressed sequence) on axial slice performed in 2012 (b) and 15 months later (c), and on coronal slice performed in 2012 (d) and 15 months later (e), have demonstrated minimal change in tumor size over time. The maximum axial dimension was taken as *X*, which is the larger of the two dimensions on axial slice (*X* and *Y*).

Tumor size measurement was acquired on both hard copies and electronic images of MRI using a standardized method (Figure 1). Dimensions were measured in 3 perpendicular axes. The largest dimensions in the anteroposterior (*X*) and mediolateral (*Y*) directions were measured on axial slices. If available, coronal or sagittal slices were utilized to measure the largest dimension in the craniocaudal direction (*Z*); otherwise it was estimated to be the product of slice thickness and number of slices in which the tumor could be identified. The maximum axial dimension was taken as *X*, which was the larger of the two dimensions on axial slice. Tumor was conceptualized to have an ellipsoid shape. The 3 perpendicular dimensions were used in the ellipsoid area and volume formulas to calculate the maximum axial area and volume of the tumor:

(1) Ellipsoid  area cm2=π12X12YEllipsoid  volume cm3=43π12X12Y12Z.

In order to standardize the measurement method on serial imaging, the baseline images were used for comparison to ensure that tumor dimensions were measured in the same orientation and on the same anatomical plane by identifying anatomical landmarks.

**Figure 2:**
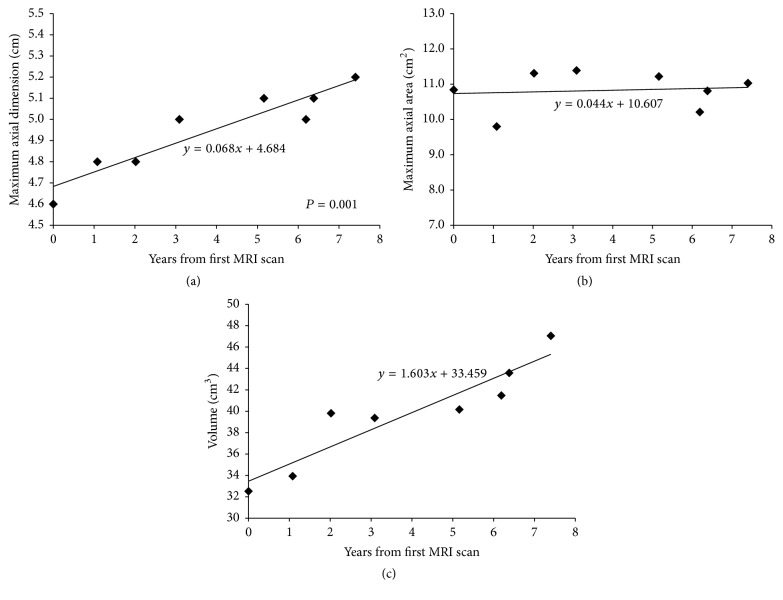
Growth rates of the untreated left glomus vagale on serial MRI scans as estimated by linear regression model. (a) Maximum axial dimension demonstrates a growth rate of 0.68 mm/year in a linear trend. (b) Maximum axial area illustrates a stable trend, with a growth rate of 0.04 cm^2^/year. (c) Volume demonstrates a growth rate of 1.6 cm^3^/year in a linear trend.

On serial MRI scans, the untreated glomus vagale demonstrated slow growth in a linear trend. No evidence of exponential growth was found. The maximum axial dimension revealed a growth rate of 0.68 mm/year (standard error 0.11 mm; *p* = 0.001; *R* ^2^ = 87%), growing from 4.6 cm to 5.2 cm (13% growth) in a linear trend (Figure 2(a)). The maximum axial area illustrated a more stable trend with a growth rate of 0.04 cm^2^/year and a minimal growth from 10.8 cm^2^ to 11.0 cm^2^ (2% growth) (Figure 2(b)). The volume demonstrated a growth rate of 1.6 cm^3^/year growing from 32.5 cm^3^ to 47.1 cm^3^ (45% growth) in a linear trend (Figure 2(c)). Tumor doubling time was estimated to be 13.82 years using the following formula [1]:

(2) $$\begin{array}{c} T_{d}=\left(\;T_{2}-T_{1}\;\right)\left[\;\frac{\left(\log2\right)}{\left(\log V_{2}-\log V_{1}\right)}\;\right], \end{array}$$

where *T*_*d*_ is tumor doubling time, *T*_2_ is last imaging time, *T*_1_ is first imaging time, *V*_2_ is volume at *T*_2_, and *V*_1_ is volume at *T*_1_.

## 3. Discussion

The current study has demonstrated slow growth of an untreated glomus vagale. Four prior radiologic studies examining the natural history of head and neck paragangliomas (Table 1) have also shown slow growth [2, 4–6]. In the existing literature, amongst tumors which have demonstrated growth, the mean growth rates for the maximum axial dimension, maximum axial area, and volume are 1.10 mm/year (range, 0.68–2.00 mm/year), 0.31 cm^2^/year (range, 0.04–0.58 cm^2^/year), and 1.00 cm^3^/year (range, 0.44–1.60 cm^3^/year), respectively. The growth rate parameters used across these studies have varied. Growth rate of the maximum axial dimension was the most widely reported growth rate parameter which was utilized in all four studies. In addition, Langerman et al. [2] have investigated the growth rate of maximum axial area. While Carlson et al. [4] and Jansen et al. [6] have examined the tumor volume utilizing the same method of volumetric measurement as our study, by acquiring 3 perpendicular tumor dimensions and using an ellipsoid volume formula to calculate the tumor volume. The current study is the first study to analyze all three growth rate parameters including the maximum axial dimension, maximum axial area, and volume. The study by Jansen et al. [6] was the only study to analyze the tumor doubling time which was 10.15 years for a cohort of 48 head and neck paragangliomas. This figure is comparable to our tumor doubling of time of 13.82 years.

Parallel to head and neck paragangliomas, vestibular schwannomas have been shown to demonstrate a slow growth. There have been many more radiologic studies evaluating the natural history of vestibular schwannomas than head and neck paragangliomas [7, 8]. Martin et al. [9] revealed a large number of conservatively managed vestibular schwannomas (78%) not showing any evidence of growth for many years following diagnosis. The growing tumors demonstrated slow growth with a mean growth rate of 4 mm/year. Several authors have recommended a “wait and scan” policy for small- or medium-sized vestibular schwannomas until growth is detected [7, 8, 10]. Likewise, in head and neck paragangliomas, the low growth rates as shown by the current report and the four existing studies may justify a similar conservative approach [2, 4–6].

The management of head and neck paragangliomas remains controversial. Boedeker [11] described the factors that play an important role in the search for optimal therapy including size, classification, and site of the tumor, and age and general health of the patient, as well as associated CN deficits. The patient's psychological and social status, treatment preferences, and baseline quality of life also need to be taken into consideration [12]. It has gradually become accepted by many clinicians that conservative management can represent effective management strategy to stand next to surgical resection and is not merely a compromise therapy reserved for those unfit for surgery. Due to the low risk of malignancy and slow growing nature of head and neck paragangliomas, it may be appropriate in selected cases to withhold any kind of invasive therapy. Observation by a “wait and scan” policy involving long-term clinical and radiologic follow-ups may be considered in asymptomatic cases [13–15]. It could also be the primary option for defining the growth pattern. However, provided the increasing life span, even slow growing tumors may progress in the long term and cause delayed and irreversible complications. Patients undergoing watchful waiting should be informed that many tumors continue to grow, and they may eventually require treatment [12]. By postponing surgery until CN impairment becomes evident or other vital structures are threatened, CN function can frequently be preserved until later in the natural history, whereas performing surgery upon diagnosis can often lead to CN impairment earlier in the history of the disease [1].

The management of glomus vagale follows the same evolving trend as head and neck paragangliomas. The decision between surgery and watchful waiting is based on critical factors including the likely natural history, age of the patient, size and growth rate of the tumor, and associated CN deficits [16]. There has been an increasing trend for glomus vagale to be managed by a “wait and scan” policy. Our finding of a slow growing glomus vagale further supports this trend. Bradshaw and Jansen [17] noted a rise in the percentage of glomus vagale undergoing watchful waiting over the years. A conservative approach in a glomus vagale patient with functional larynx is crucial, as gradually developing vagus nerve palsy may undergo satisfactory compensation. On the other hand, surgery carries the risk of aspiration following sudden loss of vagal function.

The current report is the first study in the literature to analyze the growth of an untreated glomus vagale in depth using linear regression model. In head and neck paragangliomas, the natural history is one of the critical factors which influence treatment planning. By reporting a detailed growth rate analysis of a slow growing glomus vagale has allowed better understanding of its natural history. This result also adds to the existing literature allowing clinicians to more effectively counsel head and neck paraganglioma patients as to the benefits of a “wait and scan” policy.

## Figures and Tables

**Table 1:** Literature review of radiologic studies investigating natural history of head and neck paragangliomas.

Study	Number of cases	Tumor subsite	Average FU duration (years)	Imaging	Average growth rates in growing tumors			Tumor doubling time (yr)	Number of tumors with change in size		
					Maximum axial dimension (mm/yr)	Maximum axial area (cm^2^/yr)	Volume (cm^3^/yr)		Regression	Stable	Growth
Current study	1	GV	7.5	MRI	0.68	0.04	1.60	13.82	NA	NA	1
Carlson et al., 2015 [4]	12	GJ	7.2	MRI	0.80	NA	0.44	NA	0	7 (58%)	5 (42%)
Prasad et al., 2014 [5]	23	GJ	5.1	MRI	NA^*∗*^	NA	NA	NA	3 (13%)	12 (52%)	8 (35%)
Langerman et al., 2012 [2]	47	GV (19 cases) CBT (28 cases)	5.0	CT, MRI	2.00	0.58	NA	NA	9 (20%)	19 (42%)	17 (38%)
Jansen et al., 2000 [6]	48	GV (17 cases) GJ (11 cases) CBT (20 cases)	4.2	CT, MRI	0.83	NA	NA^*∗∗*^	10.15	NA	NA	29 (60%)
Mean	26	NA	5.8	NA	1.10	0.31	1.00	11.99	11%	51%	44%^*∗∗∗*^

FU: follow-up, yr: year, GV: glomus vagale, MRI: magnetic resonance imaging, NA: not available, GJ: glomus jugulare, CBT: carotid body tumor, and CT: computed tomography.

^*∗*^An exact average value was not available. Grow rates were <3.00 mm/year in 7 tumors and >3.00 mm/year in 1 tumor.

^*∗∗*^Tumor volume was measured in this study; however the growth rate of the volume was not available.

^*∗∗∗*^This mean percentage does not include the current study which involves a single case of tumor.
